# Systematic review and meta-analysis of tumour microsatellite-instability status as a predictor of response to fluorouracil-based adjuvant chemotherapy in colorectal cancer

**DOI:** 10.1007/s00384-021-04046-x

**Published:** 2021-10-22

**Authors:** Nikhil Aggarwal, Alberto Quaglia, Mark J. W. McPhail, Kevin J. Monahan

**Affiliations:** 1 Internal Medicine, St Thomas’ Hospital, London, United Kingdom; 2 Department of Cellular Pathology, Royal Free London NHS Foundation Trust, London, United Kingdom; 3 UCL Cancer Institute, University College London, London, United Kingdom; 4 Institute of Liver Studies, Kings College London, London, United Kingdom; 5 Imperial College London, London, United Kingdom; 6 Lynch Syndrome & Family Cancer Clinic, St Mark’s Hospital, London, United Kingdom

**Keywords:** Colorectal cancer, Genetics, Chemotherapy, Oncology, Gastroenterology

## Abstract

**Purpose:**

Colorectal cancer (CRC) can be classified according to the chromosomal-instability pathway (a microsatellite-stable (MSS) pathway) and the microsatellite-instability (MSI) pathway. Adjuvant therapy after surgery in advanced CRC is usually based on fluoropyrimidine 5-fluorouracil (5-FU) alone or combined with other agents. Controversy however remains on the use of 5-FU-based regimens in treating MSI-related tumours.

**Aims:**

To systematically investigate the relationship between tumour microsatellite profile and 5-year overall survival in patients with CRC treated with 5-FU.

**Methods:**

A systematic literature review of PubMed and Embase databases was conducted. Pre-specified criteria determined study inclusion/exclusion. The PRISMA and QUADAS-2 criteria were used to assess study suitability and quality respectively. Patients were categorised as having either MSI or MSS CRC. Overall 5-year survival was estimated from Kaplan–Meier curves. Publication bias was assessed using funnel-plots and Egger’s test.

**Results:**

1807 studies were identified, with meta-analysis performed using nine studies. 5-FU treated individuals with CRC who died at 5 years were found to be 0.31 times less likely to have MSI than those who were alive, although this was not statistically significant. There was an insufficient number of studies to enable subgroup analysis by stage.

**Conclusions:**

In this meta-analysis, MSI status does not alter 5-year survival of patients with CRC patients treated with adjuvant 5-FU, however there is significant heterogeneity in the design of individual studies in the data synthesis. More studies are necessary to clarify whether CRC patients with MSI CRC, in particular early stage, should be offered 5-FU based adjuvant chemotherapy.

## Introduction

Colorectal cancer (CRC) may be classified according to the molecular pathways behind its pathogenesis and progression [1]. The molecular architecture of colorectal cancer has been largely described as two predominant pathways: the chromosomal-instability pathway accounting for approximately 85% of cases and the microsatellite-instability (MSI) pathway in the remaining 15% of cases. The MSI pathway is related to defective DNA mismatch-repair (MMR). The DNA MMR repair system controls the accuracy of DNA replication by repairing errors such as base substitutions and insertion-deletion occurring during DNA replication.

In the context of defective MMR (dMMR), repetitive DNA sequences are naturally prone to replicative errors (microsatellites). Loss of MMR protein expression may be demonstrated in tumoral tissue by immunohistochemistry. On the other hand MSI is a PCR-based assay which demonstrates the downstream effect of defective repair of DNA mismatches in cancer. Samples are usually tested for MSI by PCR using a panel of microsatellite markers. Non-neoplastic tissue is used as normal control.

Adjuvant therapy is commonly offered to patients after surgery for CRC on the grounds that it may improve the 5-year survival. Alone or combined with other agents, 5-FU has been the mainstay of adjuvant therapy for CRC and has been investigated extensively in terms of potential factors to influence its response or resistance, including MMR status.

Patients with MSI tumours have a more favourable prognosis than their MSS counterparts. This is in contrast to variable reports on the effect of 5-FU on MSI tumours. Early small non-randomized studies show a benefit, but subsequent studies showed no benefit or even a detrimental effect [2]. A seminal study published in 2003 showed that 5-FU -based adjuvant chemotherapy is not associated with a significant increase in overall and disease-free survival of patients with MSI-related CRC, and may decrease their survival [3]. There is, however, on-going controversy about the available evidence on the use fluorouracil-based regimens in treating MSI-related tumours, particularly in relation to the effect of patient’s age and in combination with other drugs.

The aim of this study was therefore to investigate, by performing a systematic review and meta-analysis, the relationship between MSI and response to 5-FU based adjuvant chemotherapy in terms of 5 year overall survival, in CRC patients.

## Methods

The Preferred Reporting Items for Systematic Reviews and Meta-analyses (PRISMA) guidelines and checklist were followed to carry out the literature review and meta-analysis of this study [4]. The article quality rating was carried out as described by Webber et al. based on the QUADAS-2 (Table 1) [5, 6].

**Table 1:** Assessment of study quality. Adapted from Webber et al. [5]

Quality rating questions	Quality categories
• Were the test(s) clearly described (number of loci tested, MMR genes, etc.) • Was the spectrum of patients/tumors representative of the patients/tumors who will receive the test in practice? • Was the patient (sample) selection process from the source population (retrospective studies) clearly described? If prospective, were patient selection criteria clearly described? • In a retrospective study, were selected samples representative (50% of original sample number; not statistically different on key characteristics e.g. stage distribution) of the original complete sample set? • Were patient withdrawals (prospective) or sample losses (retrospective) from the source population explained? • Were un-interpretable, indeterminate, or intermediate test results reported? (Includes samples with insufficient DNA) • If prospective, was treatment assignment blinded to MSI status?	• Good: Studies with a low risk of bias and minimal concerns of applicability • Fair + : Studies with some risk of bias or concerns regarding applicability; testing does meet NIH standards • Fair -: Studies with some risk of bias or concerns regarding applicability; testing does not meet NIH standards • Poor: Studies with a significant risk of bias or greater concerns regarding applicability

### Literature search

**Fig. 1:**
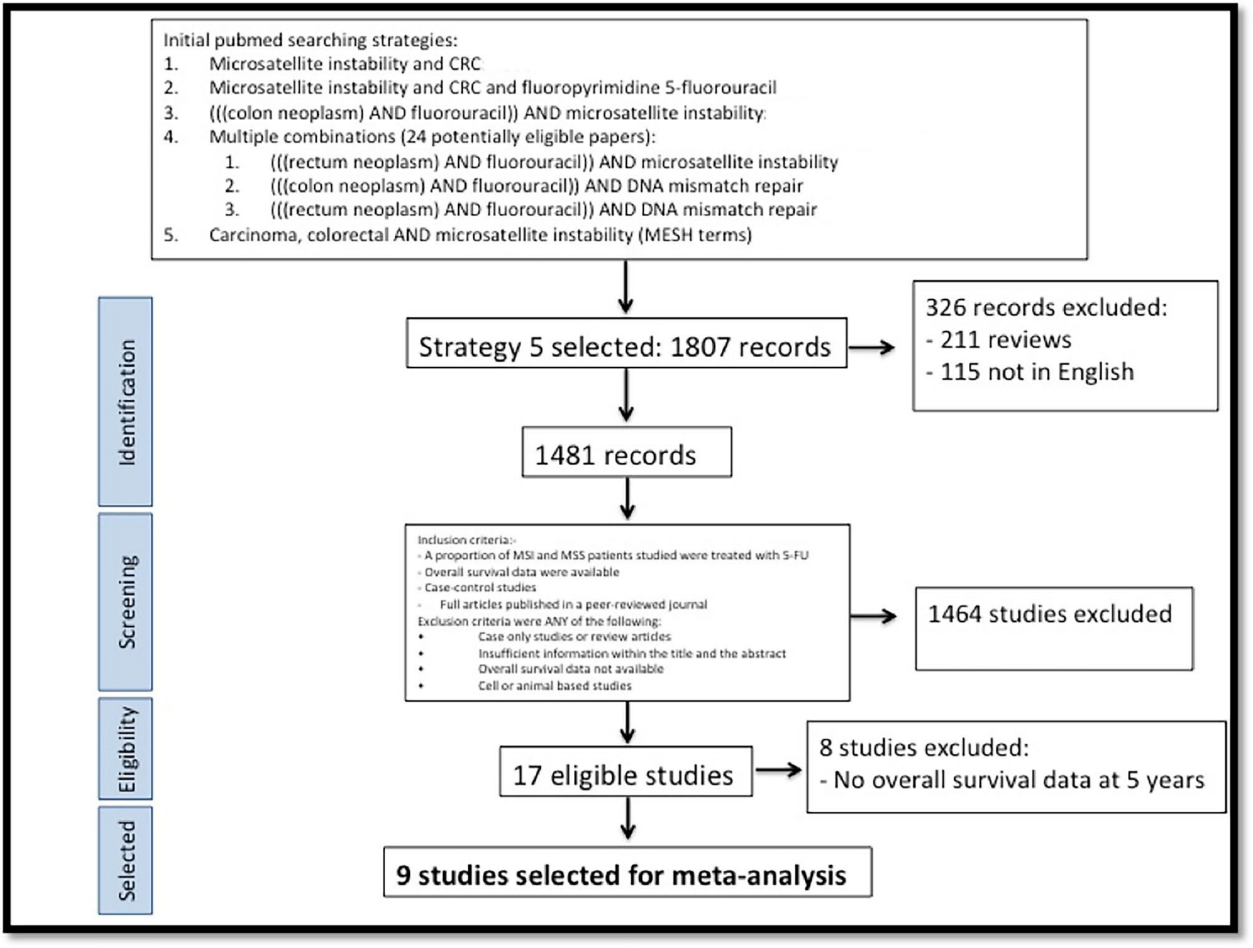
Flowchart showing selection process for studies

The literature search (Fig. 1) was carried out using the PubMed and Embase databases from conception until June 2021. Potentially informative papers were identified by reading the title of each article, and subsequently the abstract. The literature search was carried out using a combined approach because an initial searching strategy based on combinations of commonly used terms relevant to this study resulted in a number of studies too large or too small. The combination search was based on the following terms: (((colon neoplasm) AND fluorouracil)) AND microsatellite instability; (((rectum neoplasm) AND fluorouracil)) AND microsatellite instability; (((colon neoplasm) AND fluorouracil)) AND DNA mismatch repair; (((rectum neoplasm) AND fluorouracil)) AND DNA mismatch repair.

The final search was carried out by reviewing the titles of papers obtained from the combined search of "microsatellite instability"[MeSH Terms] AND "colorectal neoplasms"[MeSH Terms]” to ensure that no potentially informative papers were omitted for the initial screening process. Two independent reviewers (N.A and A.Q.) assessed the search strategy results. A strict inclusion/exclusion criteria was used to assess study eligibility (Table 2). No additional papers were identified after searching Embase.

**Table 2:** Inclusion/exclusion criteria used to assess study eligibility

Inclusion Criteria	Exclusion Criteria
Patients with CRC	Case reports or review articles
Tumour samples were tested for microsatellite instability	Insufficient information within the title and the abstract
A proportion of MSI and MSS patients studied were treated with 5-FU	Overall survival data not available
Overall survival data was available	Cell or animal based studies
Randomised clinical trial, case–control studies, cohort studies or case series	Controls with known adenomas or polyps
Full articles published in peer-reviewed journals	Insufficient information within the article for inclusion/exclusion to be established
Articles written in English	Articles not written in English

If multiple studies included the same cohort of cases or controls, the study with the largest sample size was used. In the case of any uncertainty regarding study inclusion, another investigator (K.M.) was consulted to assess eligibility.

A total of 17 papers was identified from the screening process. The full text of these 17 selected studies was accessed and retrieved for the next stage of the review. The bibliographies of relevant studies were inspected for further eligible studies.

The number of 5-FU treated patients who showed overall survival at 5 years, both in the MSI and MSS was clearly provided in three papers [3, 7, 8]. The corresponding authors of the 14 remaining papers were emailed, requesting raw data of the number of 5-FU treated MSI and MSS patients alive at five years.

A second review of the 17 eligible papers showed that 9 papers included the overall survival in the Kaplan–Meier curve and also specified the initial number of patients, for both MSI and MSS groups. The survival data were extracted using the digitizing software, digitizeIt. Briefly, survival curves from each paper where copied from the PDF document using the snapshot tool, pasted into a power point document, saved as JPEG files and opened with digitezeIt. Using the digitezeIt toolbar x and y buttons, two points on the x-axis and two points on the y-axis of each figure were marked with the cursor and their corresponding x- and y-values as indicated in each survival curve were entered. The next step involved a combination of the three digitizeIt tools, namely the point and click tool, the automatic line-selecting tool or the automatic symbol finder. The advantage of the automatic line-selecting tool is that it allows, by clicking on any point of a survival curve line, the selection of the entire line. The software returns the list of numerous line points each ordered according to its position on the line and listing each point coordinates according to the values allocated to x- and y-axis. Scrolling down the list of retrieved points identifies the 5-year mark. Its corresponding y-value indicates the survival percentage point. As the papers included the number of patients in each MSI and MSS group at the beginning of the study, the proportion of patients obtained from the Kaplan–Meier curve was used to calculate the number of patients alive at 5 years.

### Data Extraction

The following data was obtained from the papers selected for the study: study title, first author, publication year, total number of patients with CRC treated with 5-FU and of these the number of patients with MSI tumours and the number of patients with MSS tumours, and tumour stage at the time of diagnosis and treatment.

### Statistical analysis

Given that (A) cases and (A) controls correspond to the number of 5-FU treated MSI patients dead at 5 years and alive at 5 years and (B) cases and (B) controls correspond to the number of 5-FU treated MSS patients dead at 5 years and alive at 5 years, the pooled Odds Ratios (OR) were calculated as follows:

$$O.R.=\frac{\left({\displaystyle\frac{n\left(A\right)\;cases}{n\left(A\right)\;controls}}\right)}{\left({\displaystyle\frac{\left(n\left(B\right)\;cases\right)}{\left(n\left(B\right)\;controls\right)}}\right)}$$

The related 95% confidence intervals (CI) were also calculated and considered to be statistically significant if they did not intersect with 1.

The heterogeneity between studies was investigated by Cochrane’s Q statistic [9]. The I^2^ test was also used to determine whether the variation between studies was due to chance [10]. According to the I^2^ test, values range from 0 (indicating homogeneity) to 100% (indicating hetrogeneity). Heterogeneity can be graded as low (25%), medium (50%) and high (75%) according to Higgins et al. [10].

The DerSimonian and Laird random effects method was applied to generate pooled ORs if the I^2^ values were between 50 and 100% [11, 12]. The Mantel–Haenszel fixed effects test was used for I^2^ values between 0 and 50% [11, 13].

Each individual study was also removed and the analysis performed on the rest of the studies, in order to establish bias by a single study on the overall results (sensitivity analysis).

Funnel plots were used to assess publication bias. An asymmetrical funnel plot would indicate publication bias, which would be quantified using Egger’s test, taking into consideration the recommendations by Sterne et al. [14, 15].

Subgroup analyses according to cancer stage depended on a sufficiently informative number of studies with distinct stage grouping (at least 3 studies per subgroup).

The Metafor package in R (Version 3.2.4) was used for statistical analysis [16].

## Results

The selected nine papers (Table 3) included a total of 3051 patients, 2614 of whom had MSS tumours (85.7%) and 437 had MSI tumours (14.3%). Their quality and risk of bias were considered fair. Most patients were male but the ethnicity of the patient cohort was not specified in most studies. Patient’s age was variable and ranged from 20 to 85 years.

**Table 3:** Summary of the studies used for the meta-analysis

Author	Year	Type of study	Patient details					Median follow-up	Tumour details	Molecular method	Tissue details
			Total number of patients	Male/female	Age (years)	Ethnicity	MSI/MSS in 5-FU treated patients				
Ribic (3)	2003	Retrospective MSI study of samples from patients enrolled in multicentre US/Canada phase 3 randomized controlled trials of adjuvant chemotherapy: - 5-FU plus Leucovorin or no treatment (3 studies) - 5-FU plus levamisole or no treatment (2 studies)	570	-326 male (57%) -244 female (43%)	59.8 ± 11.2	No details given	MSI: 53 MSS: 230	7.4 years	Colon Stage II-III Grading: well to undifferentiated Histotype: not specified	PCR Variable number of microsatellite loci (2–11)	At least 60% tumour cells
Hong (21)	2012	Retrospective single centre case control study from Republic of Korea comparing: -Surgery alone -Surgery plus 5-FU -Surgery plus 5-FU and radiotherapy	1125	-691 male (61.4%) -434 female (38.6%)	61 (26–92)	No details given	MSI: 81 MSS: 791	3.5 years	Colon-rectum TNM stage I to IV Grading: well to undifferentiated Histotype: not specified	PCR 5 microsatellite loci (Bethesda panel)	Not given
Jover (24)	2009	Nested prospective follow-up multicenter study, 10 hospitals in Spain Comparison of 5-FU adjuvant vs. surgery alone	754	-456 male -298 female	MMR proficient: 70.0 (11.5) MMR deficient: 68.4 (13.8)	No details given	MSI: 26 MSS: 225	4.1 years	Tumour site not specified TNM Stage I-IV Grading: not specified Histotype: not specified	PCR 5 microsatellite loci (Bethesda panel) ABI 310 Genetic Analyzer or immunohistochemistry (MLH1 or MSH2)	Not given
Kim (18)	2007	Retrospective MSI testing of patients drawn from four US clinical trials (1983–1990): - Surgery alone arm from 2 trials - 5-FU and Leucovorin from 2 trials	1044	No details given	No details given	No details given	MSI: 61 MSS: 308	Not given	Colon Dukes B and C Grading: not specified Histotype: not specified	PCR 5 microsatellite loci (Bethesda panel) ABI 310 Genetic Analyzer	At least 50% of tumour cells
Carethers (19)	2004	Case control retrospective study of patients from two hospitals in San Diego California treated with or without 5-FU adjuvant therapy	204	-131 male -73 female	65.73 _ 13.5	Asian 6% Black 7% Hispanic 10% White 76% Unknown 1%	MSI: 10 MSS: 56	3.6 years	Colon Stage II and III Grading: well to poor Histotype: -Mucinous 60 - Non mucinous 117	PCR 5 microsatellite loci (Bethesda panel) Polyacrylamide gel	Not given
Ohrling (22)	2010	Retrospective MSI testing on patients enrolled in a randomized clinical trial 1991–1996 on surgery with or without 5-FU (plus-minus levamisole). Multicentre from 59 hospitals in Sweden	1006	-559 male -447 female	66 (24–75)	No details given	MSI: 72 MSS: 274	Not given	Colon-rectum Stage II and III Grading: well to poorly differentiated or unknown Histotype: not specified	MLH1 and MSH2 immunohistochemistry	Not applicable
Klingbiel (8)	2015	MSI testing on patients enrolled in a randomized phase III clinical trial 2000–2002 multicentre (31 countries) on 5-FU or FOLFIRI	1254	No details given	MSI: 54 (25–75) -MSS: 61 (21–76)	No details given	MSI: 45 MSS: 160	5.75 years	Colon TNM stage I to IV Grading: well to poorly differentiated Histotype: mucinous 81% Non mucinous 18% Unknown 1%	PCR 5 microsatellite loci (Bethesda panel) plus (BAT-25, BAT-26, D2S123, D5S346, TGFBR2, BAT-40, D17S787, D18S69, D17S250, and D18S58)	Not given
Bertagnolli (20)	2009	Randomised control trial phase III randomized trial comparing FU/ Leucovorin (LV) with irinotecan, FU, and LV (IFL) for postoperative adjuvant treatment. Two centres Canada and US	1264	-Male 702 -Female 562	61 (21–85)	No details given	MSI: 46 MSS: 302	6.65 years	Colon TNM stage III Grading: well to poorly differentiated or unknown Histotype: mucinous 9.9% Non mucinous 85.1% Unknown 5%	MLH1 and MSH2 immunohistochemistry and PCR 5 microsatellite loci (Bethesda panel) No details on analysis	At least 60% of tumour cells using microdissection when necessary
Jensen (23)	2009	Retrospective MSI on a cohort of 5FU adjuvant treated patient in a single hospital, 1996–2003, Copenhagen	311	-Male 159 -Female 152	-MSI: < 70 = 28; > 69 = 15 -MSS: < 70 = 217 > 69 = 51	No details given	MSI: 43 MSS: 268	6.1 years	Colo-rectal TNM stage II-IV Grading: well to poorly differentiated Histotype: not specified	MLH1, MSH2, MSH6 and PMS2 immunohistochemistry and PCR 5 microsatellite loci (Bethesda panel) No details on analysis	At least 50% of tumour cells

Most studies referred to the previously published National Cancer Institute panel for the definition of MSI as follows: ‘high frequency MSI as instability at 30% or more of the screened loci, low frequency instability as less than 30% of the loci screened and microsatellite stability as stability at all loci screened’ [17]. Tumours with low frequency microsatellite instability were generally considered to be biologically similar to those with microsatellite stability and were grouped together.

In five studies the tumours were located in the colon [3, 8, 18–20]. Three studies included a combination of colonic and rectal tumours [21–23]. In one study the precise location of the tumours investigated was not specified [24]. There was no specific mention in most of these papers whether any of the patients had Lynch syndrome.

The results of the 5-year survival analysis are shown in Table 4. In all studies the number of MSS patients was higher than MSI patients in keeping with the known predominance of chromosomal instability tumours in the general population.

**Table 4:** Results of 5-year survival in MSI and MSS patients with CRC treated with 5-FU

Author	MSI Dead	MSI Alive	MSS Dead	MSS Alive	Total MSI	Total MSS
Ribic (3)	18	35	57	173	53	230
Hong (21)	7	74	215	576	81	791
Jover (24)	7	19	54	171	26	225
Kim (18)	14	47	75	233	61	308
Carethers (19)	1	9	20	36	10	56
Ohrling (22)	20	52	97	177	72	274
Klingbiel (8)	1	44	16	144	45	160
Bertagnolli (20)	15	31	84	218	46	302
Jensen (23)	9	34	109	159	43	268

As the I^2^ value was 64.87%, indicating moderate to high heterogeneity, the random effects model was used to generate the forest plot.

**Fig. 2:**
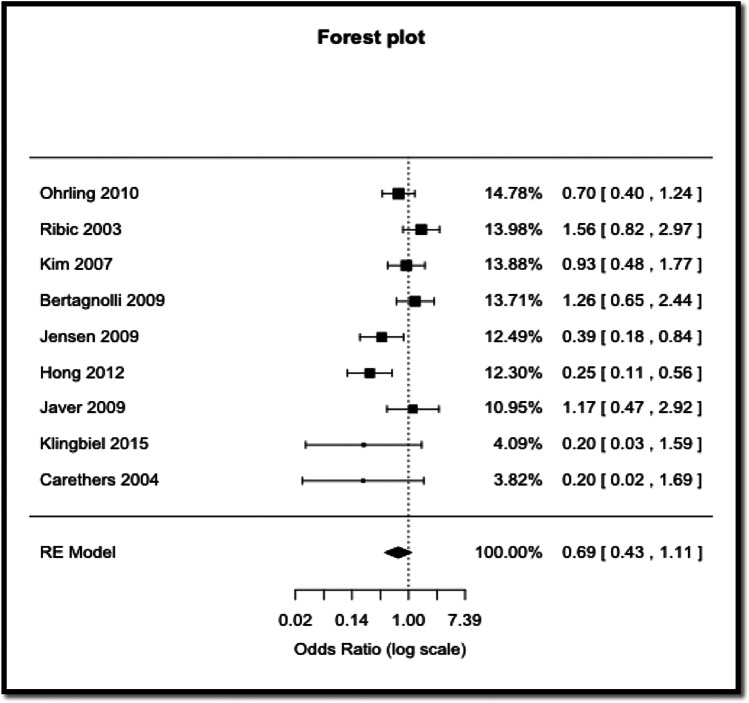
Forest plot of MSI vs. MSS in 5-FU treated CRC patients

The forest plot for displayed pooled ORs with 95% CI as well as study weightings Is shown in Fig. 2. According to the meta-analysis result, 5-FU treated individuals who died at 5 years were 0.69 times as likely to have MSI, rather than MSS, compared to those who were alive, although this did not reach statistical significance.

**Fig. 3:**
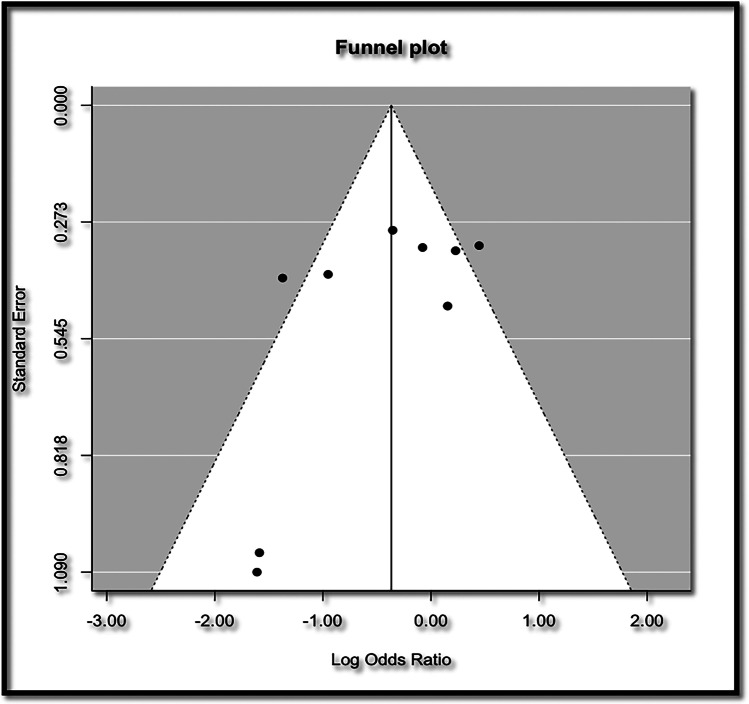
Funnel plot of studies

In terms of publication bias, the Funnel plot was symmetrical confirming that there was no publication bias (Fig. 3) and this is supported by the Eggers test, which was not significant (t = -1.3784, df = 7, p = 0.2105). Subgroup analyses according to cancer stage were not conducted because there were an insufficient number of informative studies with distinct stage grouping.

## Discussion

The aim of this study was to perform a systematic review and meta-analysis to investigate whether microsatellite instability influences 5-year survival in CRC patients treated with 5-FU. The seminal paper by Ribic et al. showed a better survival rate in MSI CRC patients not treated by adjuvant 5-FU therapy; a better survival rate in MSS patients treated by adjuvant 5-FU therapy; and no benefit in providing adjuvant 5-FU therapy to MSI patients [3]. The result of this meta-analysis shows that 5-FU treated CRC patients who died at 5 years were 0.31 times less likely to have MSI tumours, rather than MSS tumours, compared to those who were alive, although this difference did not reach statistical significance. This result broadly confirms two previous meta-analysis studies [2, 5].

Highly conserved from prokaryotes to eukaryotes, the DNA mismatch repair machinery is based on the assembly of the MSH2, MSH3 and MSH6 proteins to make the two heterodimers MutSa (MSH2/MSH6) and MutSb (MSH2/MSH3). MutSa or MutSb form a ternary complex with MutLa (composed of the two other proteins MLH1 and PMS2), and together with other proteins such as PCNA and RPA, repair replication errors [25].

In most MSI CRCs, dMMR is due to epigenetic hypermethylation of CpC islands located in the promoter region of the *MLH1* gene and diffuse hypermethylation of CpG dinucleotides in the promoters regions of *TP16* and *CDH1* tumour suppressor genes. These hypermethylation events are called high frequency CpG island methylator phenotype (CIMP-H). Almost all *MLH1* hyper methylated CRCs have CIMP-H [26].

5-FU metabolites are active and have two main actions: a) inhibition of the enzyme nucleotide synthetic enzyme thymidylate synthase (TS); b) incorporation of fluoronucleotides into RNA and DNA. TS catalyses the conversion of deoxyuridine monophosphate (dUMP) to deoxythymidine monophosphate (dTMP), which is essential for DNA replication and repair. Many factors have been shown to correlate with the effect of 5-FU based chemotherapy. Not surprisingly, low TS expression in tumour cells is associated with higher response to 5-FU, which may also depend on TS promoter variants [2]. Deficient hepatocytes can result in 5-FU toxicity and p53 overexpression has been shown to be associated with resistance to 5-FU [18]. There is in vitro evidence that MSI cell lines are resistant to 5-FU, and that biallelic hypermethylation of hMLH1 eliminates 5-FU resistance. Resistance by MSI tumour cells to 5-FU could also be due to a direct interaction between MMR proteins and 5-FU and its metabolites and the effect of other factors such a p53 [2].

We recognise our study has limitations. Tumour staging was variable in the selected studies. It was not possible to perform a subgroup analysis according to tumour stage due to the limited information available and the limited number of informative studies. For example, some studies did not clarify which staging system had been used, and could not be compared to others. Staging remains a relevant criterion to guide adjuvant 5-FU based chemotherapy in CRC patients.

There was insufficient information overall on tumour histology. More aggressive subtypes (e.g. mucinous, undifferentiated) can influence prognosis, although this effect may be overrun by MSI status. Histological criteria for the diagnosis, and staging of CRC have evolved over the years, and therefore data acquired at an interval of 10 to 15 years may not be fully comparable.

As noted in previous meta-analysis studies, the limited number of MSI patients results in larger confidence intervals and a reduced statistical power when compared to data obtained from MSS patients [2, 5]. Of the MSI CRCs, approximately 10–13% are sporadic, and 2–5% occur in the context of a Lynch syndrome. Patients with Lynch syndrome are at increased risk of CRC as well as cancers at other sites including endometrial, ovarian, gastric, and hepato-biliary pancreatic cancers. There was no mention of Lynch syndrome in most papers selected for this meta-analysis.

Most studies adhered to the Bethesda panel for the assessment of microsatellite instability [26]. The methods used however were variable. The number of microsatellite loci investigated ranged from 2 to 11, probably in line with changes in criteria over the years. The core panel recommended by the National Cancer Institute workshop in 1997 consisted of two mononucleotide repeats (*BAT25, BAT26*) and three dinucleotide repeats (*D5S346, D2S123, D17S250*) [27]. The revised Bethesda panel in 2002 included additional mononucleotide markers because the use in the original panel of three dinucleotide repeats could underestimate the number of MSI tumours whereas the use of two mononucleotide repeats could overestimate the number of MSI-L tumours (where < 40% of microsatellites demonstrate instability in the panel) [28].

The proportion of tumour cells present in the samples used for MSI testing was mentioned in four studies only, and was at least 50% in two and 60% in the other two. The College of American Pathologists (CAP) MSI proficiency survey of 104 US laboratories showed that an insufficient tumour content in samples used for MSI testing could have been responsible for some misclassified cases when results of different laboratories were compared [29]. Use of microdissection resulted in the reduced rate of misclassified cases, and there was a significant difference in the rate of MSI tumours between the laboratories that used and those that did not use microdissection. This survey also highlighted the lack of consensus on the minimum amount of tumour cellularity necessary for reliable MSI testing, the reported requirement ranging from 11 to 40%. Approximately 10% of tumour cells was the minimum requirement for identifying MSI in a study based on serial dilutions of a microdissected specimen [30]. Laboratories testing for MSI should therefore mention the risk of a false negative result when using suboptimal samples. The interobserver variability in assessing the percentage of neoplastic cells in tissue samples is, however, significant. There is a tendency to overestimate when an overall estimate is compared with a cell counting method. Overestimating tumour cellularity carries the risk of increasing the number of false negatives. A small proportion of the surveyed laboratories used laser-capture microdissection to isolate tumour cells for DNA extraction.

The studies selected in this meta-analysis provided 5-year survival data as Kaplan–Meier curves for 5-FU treated MSI and MSS patients. Survival at different time points can be extracted from survival curves, as noted by Duchateau and colleagues [31]. The approach used in this study is a compromise between the complex techniques of both Guyot et al. and Liu et al. and the more traditional ‘pencil and ruler’ approach to ‘read off survival probabilities’ [32, 33]. The main limitation of this approach, however, is that it does not consider censoring. In our series, we considered the estimated percentage of patients alive at 5 years relatively high ranging from 66 to 98% (average 79%) in the MSI patients and from 59 to 90% (average 72%) in the MSS patients. We also made the assumption that by using the same approach in all the nine selected papers, as opposed to combining data extracted from Kaplan–Meier curve with raw data provided in table format, the effect of censoring would be minimised and data would be comparable. It is for this reason that we decided not to include, in this series, the OR generated by the raw data available in the paper by Sargent et al. and to extract the data from the Kaplan–Meier curves in the two papers by Ribic et al. and Klingbiel et al., despite the raw data being available in table format [3, 7, 8].

## Conclusion

This meta-analysis shows there is no significant difference in the overall survival of patients with MSI CRC and MSS CRC treated with adjuvant 5-FU. Further studies are necessary to clarify whether patients with MSI CRC, and in particular those at a relatively early stage, should be offered 5-FU based adjuvant chemotherapy. Additional investigations in the molecular pathways involved in the metabolism and function of 5-FU and its metabolites could help in identifying patients more or less responsive to 5-FU, and monitor its effects, in the context of precision medicine and pharmacogenomics.
